# Measuring e-learning systems success: Data from students of higher education institutions in Morocco

**DOI:** 10.1016/j.dib.2021.106807

**Published:** 2021-01-30

**Authors:** Abdelaziz Ouajdouni, Khalid Chafik, Omar Boubker

**Affiliations:** a Management and Information System Research Group, National School of Business & Management, Abdelmalek Essaadi University, Tangier, Morocco; b Department of Management, Laayoune Higher School of Technology, Ibn Zohr University, Morocco

**Keywords:** Covid-19, E-learning, Online learning platforms, E-learning system use, E-learner satisfaction, Learner computer anxiety, Instructor quality, PLS-SEM approach

## Abstract

The COVID-19 pandemic has forced Higher Education Institutions (HEI's) to rethink the teaching approach taken. In response to this emergency state, Moroccan universities switched to the e-learning approach as an alternative to face-to-face education. At this level the assessment of e-learning systems success becomes a necessity. This data article aims to identify e-learning systems success determinants during the COVID-19 pandemic. The data was collected from students of the Moroccan Higher Education Institutions. The research data are collected via an on a self-administered online questionnaire, from a sample of 264 university students. The responses are collected from students of 12 Moroccan universities and 31 Moroccan educational institutions. The data were analyzed using a structural equation modeling method under the Partial Least Squares approach (PLS-SEM). Data analysis was performed using SmartPLS 3 software. Universities managers can use the dataset to identify key factor to enhance e-learning system success.

## Specifications Table

**Table:** 

Subject	Education management; Management Information Systems
Specific subject area	E-learner satisfaction; E-learning systems success; Social influence
Type of data	Tables and Figures
How data were acquired	A survey was carried out among students of the Moroccan Higher Education Institutions (HEI's).
Data format	Raw, analyzed and descriptive data
Parameters for data collection	The sample consisted of students of the Moroccan Higher Education Institutions. The questionnaire was self-administered via the Google Forms tool during the months of May and June 2020.
Description of data collection	The survey link was disseminated via social networks.
Data source location	12 Universities in Kingdom of Morocco.
Data accessibility	Repository name: Mendeley Data Data identification number: http://dx.doi.org/10.17632/h9vdjh8tk7.2 Direct URL to data: https://data.mendeley.com/datasets/h9vdjh8tk7/2

## Value of the Data

• The dataset is useful because it helps to explore the factors that affect the E-Learning systems success in Higher Education Institutions (HEI's).
• This dataset can be used to enlighten Moroccan educational institutions managers on the importance of system quality and instructor quality as a key factor to improve perceived usefulness, e-learning systems use and e-learners satisfaction.
• The dataset will be useful for universities managers and policymakers to renovate practices in order to enhance e-learning system use, e-learners satisfaction, and e-learning system success.
• This dataset provides insights into diverse aspects of system quality, instructor quality, social influence, learner computer anxiety, perceived usefulness, e-learning system use, e-learner satisfaction, and e-learning system success.
• This dataset can be adapted for use in order to assess the e-learning system success in primary and secondary education.

## 1 Data Description

The constructs and measurement items used in this data article were drawn from previous research (Table 1). A questionnaire survey was carried out among Moroccan Higher Education Institutions (HEI's). The questionnaire was self-administered via the Google Forms tool during the months of May and June. The research data and questionnaire are available in Mendeley data on: https://data.mendeley.com/datasets/h9vdjh8tk7/2

**Table 1:** Measurement instruments.

Variables	Adapted Items	Source	
System Quality	SQ1	The e-learning system is easy to navigate.	[1]
	SQ2	The e-learning system allows me to easily find the information I am looking for.	
	SQ3	The e-learning system is easy to use	
Instructor Quality	IQ1	I use e-learning system as recommended by my instructors	[2] [3]
	IQ2	I think an instructor's enthusiasm about using e-learning stimulates my desire to learn	
	IQ3	I receive a prompt response to questions and concerns from my instructors in e-learning	
	IQ4	I think communicating and interacting with instructors are important and valuable in e-learning	
	IQ5	Generally, my instructors have a positive attitude to the utilization of e-learning	
Social Influence	SInf1	People who are important to me think that I should use e-learning	[4]
	SInf2	People who influence my behavior think that I should use e-learning	
	SInf3	People whose opinions that I value prefer that I use e-learning	
	SInf4	My organization supports the use of e-learning	
Learner Computer Anxiety	LCA1	Working with a computer would make me very nervous	[5]
	LCA2	I get a sinking feeling when I think of trying to use a computer	
	LCA3	Computers make me feel uncomfortable	
Perceived Usefulness	PU1	Use of the chosen e-Learning tool enabled me to accomplish tasks more quickly.	[6]
	PU2	Use of the chosen e-Learning tool improved the quality of my tasks.	
	PU3	Use of the chosen e-Learning tool enhanced the effectiveness of my tasks.	
	PU4	As a whole, the chosen e-Learning tool is useful to me.	
E-Learning System Use	ELU1	Retrieve information.	[1]
	ELU2	Publish information.	
	ELU3	Communicate with colleagues and teachers.	
	ELU4	Store and share documents.	
	ELU5	Execute course work	
	ULU6	I currently use e-learning systems (1). Not at all; (2). About once a week; (3). Four or six times a week; (4). About once a day; (5). Several times a day	[7]
E-Learner Satisfaction	ELS1	E-learning is enjoyable	[8] [9] [10]
	ELS2	E-learning give me self-confidence	
	ELS3	E-learning satisfies my educational needs	
	ELS4	I am satisfied with performance of system	
	ELS5	E-learning is pleasant to me	
	ELS6	I am pleased enough with e-learning system	
E-Learning System Success	ELSS1	The system has a positive impact on my learning	[11]
	ELSS2	Overall, the performance of the system is good	
	ELSS3	Overall, the system is successful	
	ELSS4	The system is an important and valuable aid to me in the performance of my class work.	
	ELSS5	The system helps me to Increase knowledge (increased knowledge)	[12]
	ELSS6	The system helps me to Increase Self-reliance (self-reliance)	

5-point Likert-scale: [Strongly disagree 0.1] - [.2] - [.3] - [.4] - [5. Strongly agree].

Due to the lack of a sample frame, we have resorted to a non-probabilistic sampling method. This kind of method is used for practical reasons of accessibility and reduced cost. Table 2 illustrates the profile and characteristics of students who participated in this survey. A total of 264 responses from students were received, including 187 women (70.80%) and 77 men (29.20%). Almost half of the respondents to our questionnaire are undergraduate students (46.2%). The responses are collected from students of 31 Moroccan educational institutions affiliated with 12 universities (Tables 3 and 4). 25.67% of students indicate that they do not use any video conferencing systems and 17.05% among them do not use any online learning platforms. As an alternative, teachers refer to WhatsApp groups in order to interact with students, as they use YouTube videos for transferring knowledge. It is to highlight that Google meet and Zoom are the most video conferencing systems used in Moroccan HEI's. Additionally, Moroccan students use several online learning platforms such as; Coursera, Google Classroom, LinkedIn Learning, Moodle, and Udemy (Table 5).

**Table 2:** Profile and characteristics of respondents (*n* = 264).

Attributes	Characteristic	Frequency	Percentage (%)
Gender	**Female**	**187**	70.80%
	**Male**	**77**	29.20%
Level of studies	**BAC+1**	**60**	22.70%
	**BAC+2**	**29**	11.00%
	**BAC+3**	**122**	46.20%
	**BAC+4**	**41**	15.50%
	**BAC+5**	**8**	3.00%
	**PhD Student**	**4**	1.50%

**Table 3:** Universities of the students who participated in the survey.

University	Frequency	Percentage (%)
Ibn Zohr University	157	59,47%
Abdelmalek Essaadi University	44	16,67%
Mohammed First University	28	10,61%
Chouaib Doukkali University	11	4,17%
Cadi Ayyad University	7	2,65%
Sidi Mohammed ben Abdellah University	4	1,52%
Mohammed V University	4	1,52%
Hassan First University	2	0,76%
Hassan II University	2	1,14%
Sultan Moulay Slimane University	2	0,76%
Ibn Tofail University	1	0,38%
Moulay Ismail University	1	0,38%
**Total**	**264**	**100%**

**Table 4:** Educational institutions of the students who participated in the survey.

Educational institutions	Frequency	Percentage (%)
National School of Commerce and Management of Agadir	112	42,42
National School of Commerce and Management of Tangier	44	16,67
Higher School of Technology - Laayoune	25	9,47
Higher School of Technology of Oujda	21	7,95
National School of Commerce and Management of El Jadida	11	4,17
Higher School of Technology - Agadir	8	3,03
Higher School of Technology - Guelmim	7	2,65
National School of Commerce and Management of Dakhla	4	1,52
National School of Commerce and Management of Oujda	3	1,14
Faculty of Legal, Economic and Social Sciences - Oujda	2	0,76
Faculty of Legal, Economic and Social Sciences - Salé	2	0,76
Faculty of Sciences Dhar El Mehraz - Fez	2	0,76
Higher School of Technology - Essaouira	2	0,76
Higher School of Technology - Oujda	2	0,76
National School of Applied Sciences - Khouribga	2	0,76
National School of Commerce and Management of Settat	2	0,76
Ait Melloul University Campus	1	0,38
Faculty of Legal, Economic and Social Sciences - Marrakech	1	0,38
Faculty of Legal, Economic and Social Sciences - Souissi	1	0,38
Faculty of Legal, Economic and Social Sciences of Ain Sebâa	1	0,38
Faculty of Medicine and Pharmacy - Oujda	1	0,38
Faculty of Sciences - Casablanca	1	0,38
Faculty of Sciences and Techniques of Marrakech	1	0,38
Faculty of Sciences and Techniques of Mohammedia	1	0,38
Higher Normal School of Fez	1	0,38
Higher Normal School of Meknes	1	0,38
Higher School of Technology - Fez	1	0,38
Higher School of Technology - Marrakech	1	0,38
Higher School of Technology of Essaouira	1	0,38
National School of Commerce and Management of Marrakech	1	0,38
Polydisciplinary Faculty of Larache	1	0,38
**Total**	**264**	**100,00**

**Table 5:** Video conferencing systems and online learning platforms used in Moroccan universities.

		Frequency	Percentage (%)
**Video conferencing systems**	Google Meet	93	35,23%
	Zoom	92	34,85%
	Big blue button	6	2,27%
	Cisco Webex	5	1,89%
	Other	68	25,76%
**Online learning platforms**	Coursera	47	17,80%
	Google classroom	45	17,05%
	LinkedIn Learning	41	15,53%
	Udemy	23	8,71%
	Edx	15	5,68%
	Khan Academy	10	3,79%
	Moodle	9	3,41%
	DataCamp	7	2,65%
	SKILLSHARE	7	2,65%
	FUN MOOC	6	2,27%
	MUN MOOC	4	1,52%
	Lynda.com	2	0,76%
	Easyclass	1	0,38%
	Edrak	1	0,38%
	OpenClassroom	1	0,38%
	Other	45	17,05%

## 2 Experimental Design, Materials and Methods

**Fig. 1:**
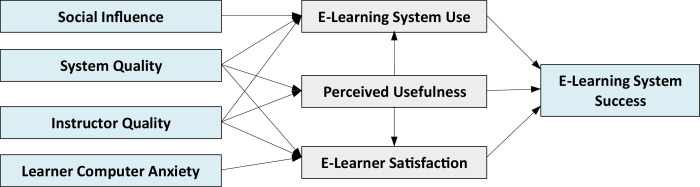
Conceptual framework.

Fig. 1 illustrates the research hypotheses based on previous research. To test the research model, we used the Partial Least Squares approach). Because of the exploratory character and the small size of our sample, we have used the PLS-SEM as an appropriate method to analyze hypothesis and research model.

**Fig. 2:**
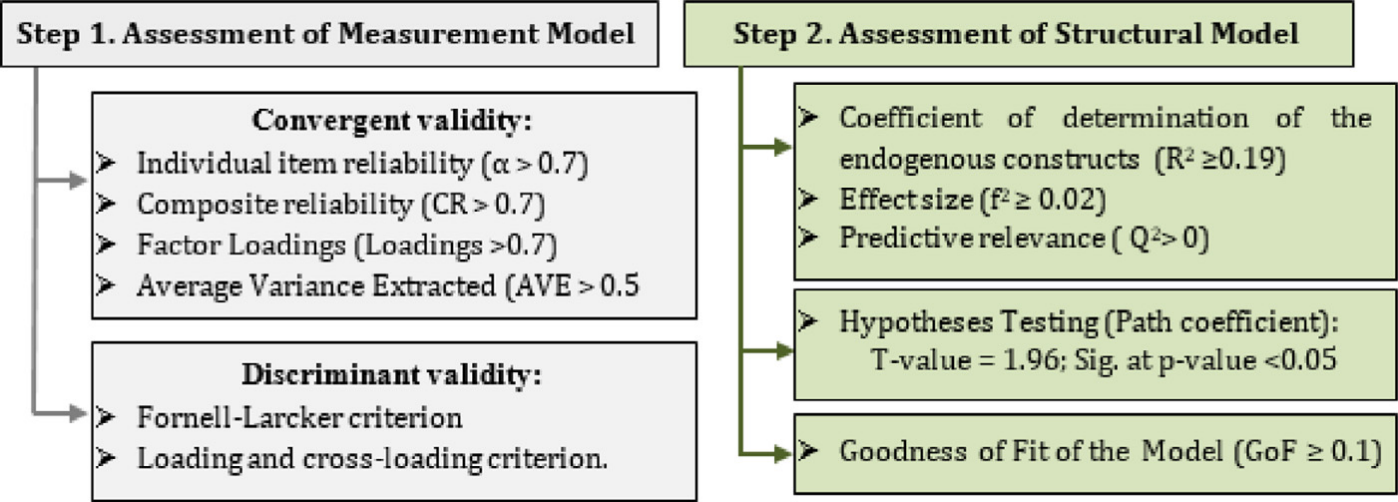
Partial least squares approach steps.

Fig. 2 summarizes steps of the structural equation modeling method under the Partial Least Squares approach [13], [14], [15].

**Fig. 3:**
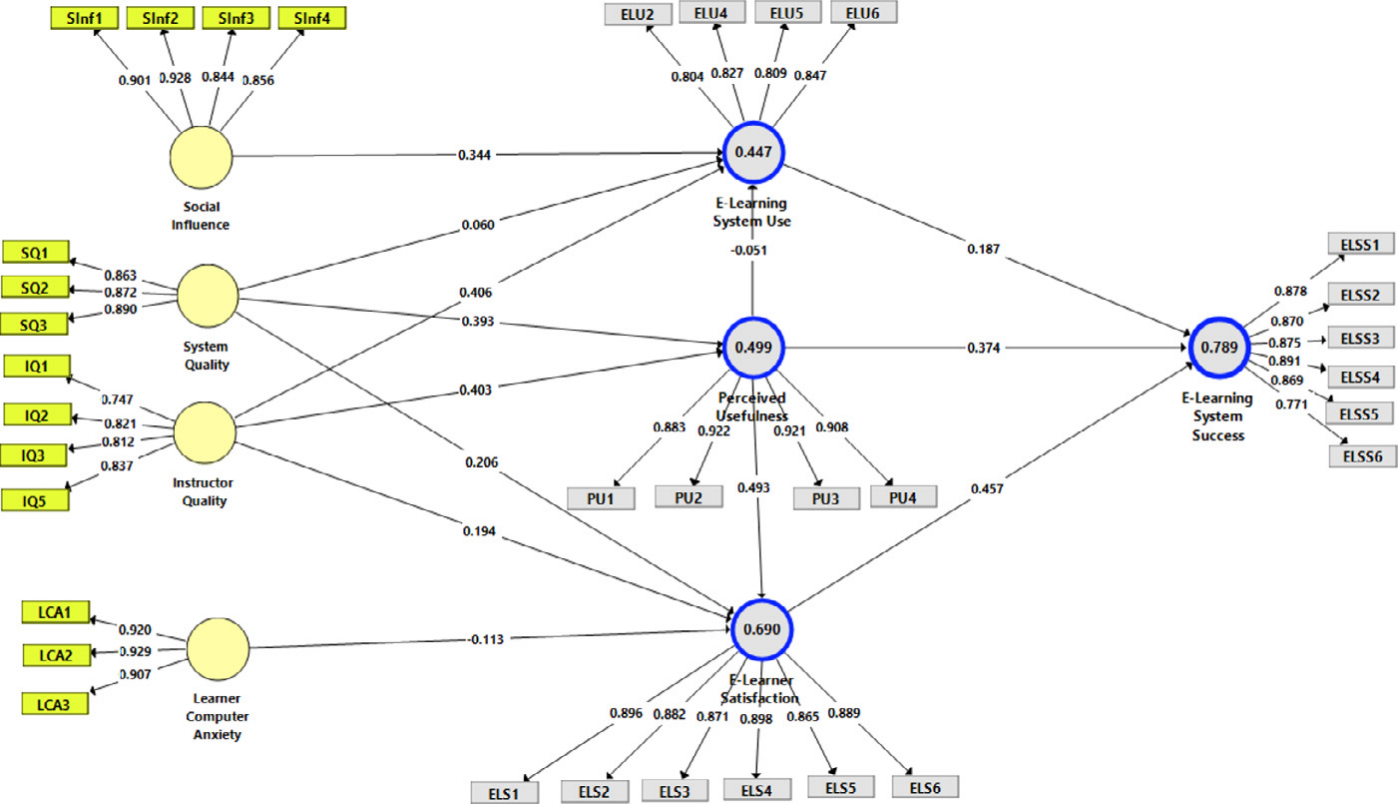
Measurement and structural model - Output SmartPLS.

For data analysis, we used the SmartPLS 3 software. Table 6 summarizes the convergent validity, according to several criteria: individual item reliability (>0.7), composite reliability (>0.7), factor loadings (>0.7) and average variance extracted (AVE>0.5). Likewise, the discriminant validity is ensured thanks to the Fornell-Larcker criterion (Table 7), and the cross-loading criterion (Table 8). In short, Fig. 3 shows the SEM-PLS estimation for the measurement and structural model.

**Table 6:** Convergent validity.

Constructs	Items	Outer loading (>0.7)	Cronbach's alpha (>0.7)	rho_A (>0.7)	CR (>0.7)	AVE (>0.5)
**System Quality** **(SQ)**	SQ1	0.863	0.848	0.852	0.908	0.766
	SQ2	0.872				
	SQ3	0.890				
**Instructor Quality** **(IQ)**	IQ1	0.747	0.819	0.824	0.880	0.648
	IQ2	0.821				
	IQ3	0.812				
	IQ5	0.837				
**Social Influence** **(SInf)**	SInf1	0.901	0.906	0.913	0.934	0.779
	SInf2	0.928				
	SInf3	0.844				
	SInf4	0.856				
**Learner Computer Anxiety (LCA)**	LCA1	0.920	0.908	0.910	0.942	0.845
	LCA2	0.929				
	LCA3	0.907				
**Perceived Usefulness** **(PU)**	PU1	0.883	0.929	0.930	0.950	0.825
	PU2	0.922				
	PU3	0.921				
	PU4	0.908				
**E-Learning** **System Use (ELU)**	ELU2	0.804	0.840	0.845	0.893	0.676
	ELU4	0.827				
	ELU5	0.809				
	ELU6	0.847				
**E-Learner Satisfaction (ELS)**	ELS1	0.896	0.944	0.945	0.955	0.781
	ELS2	0.882				
	ELS3	0.871				
	ELS4	0.898				
	ELS5	0.865				
	ELS6	0.889				
**E-Learning System Success** **(ELSS)**	ELSS1	0.878	0.929	0.933	0.944	0.740
	ELSS2	0.870				
	ELSS3	0.875				
	ELSS4	0.891				
	ELSS5	0.869				
	ELSS6	0.771

**Table 7:** Discriminant validity (Fornell-Larcker criterion).

Constructs	ELS	ELSS	ELU	IQ	LCA	PU	SInf	SQ
E-Learner Satisfaction (ELS)	0.884*							
E-Learning System Success (ELSS)	0.832	0.860*						
E-Learning System Use (ELU)	0.443	0.556	0.822*					
Instructor Quality (IQ)	0.644	0.669	0.608	0.805*				
Learner Computer Anxiety (LCA)	−0.387	−0.296	−0.085	−0.194	0.919*			
Perceived Usefulness (PU)	0.782	0.815	0.446	0.629	−0.347	0.909*		
Social Influence (SInf)	0.595	0.630	0.576	0.579	−0.218	0.595	0.883*	
System Quality (SQ)	0.661	0.670	0.418	0.574	−0.318	0.625	0.456	0.875*

⁎ Root square of AVE.

**Table 8:** Discriminant validity - loading and cross-loading criterion.

	ELS	ELSS	ELU	IQ	LCA	PU	SInf	SQ
**ELS1**	**0.896**	0.721	0.369	0.529	−0.384	0.722	0.510	0.620
**ELS2**	**0.882**	0.738	0.411	0.535	−0.356	0.716	0.569	0.561
**ELS3**	**0.871**	0.752	0.439	0.607	−0.270	0.675	0.497	0.585
**ELS4**	**0.898**	0.762	0.410	0.636	−0.346	0.700	0.514	0.613
**ELS5**	**0.865**	0.691	0.318	0.472	−0.332	0.646	0.514	0.537
**ELS6**	**0.889**	0.745	0.398	0.626	−0.363	0.686	0.548	0.582
**ELSS1**	0.794	**0.878**	0.431	0.572	−0.333	0.769	0.511	0.535
**ELSS2**	0.726	**0.870**	0.469	0.575	−0.224	0.695	0.534	0.646
**ELSS3**	0.771	**0.875**	0.451	0.596	−0.250	0.731	0.550	0.641
**ELSS4**	0.706	**0.891**	0.489	0.596	−0.261	0.700	0.550	0.547
**ELSS5**	0.698	**0.869**	0.520	0.585	−0.247	0.710	0.587	0.510
**ELSS6**	0.577	**0.771**	0.529	0.529	−0.202	0.583	0.527	0.587
**ELU2**	0.369	0.461	**0.804**	0.495	−0.073	0.363	0.498	0.411
**ELU4**	0.423	0.466	**0.827**	0.572	−0.071	0.393	0.477	0.355
**ELU5**	0.248	0.395	**0.809**	0.409	0.016	0.287	0.420	0.198
**ELU6**	0.396	0.497	**0.847**	0.506	−0.135	0.410	0.491	0.385
**IQ1**	0.397	0.460	0.551	**0.747**	−0.105	0.382	0.486	0.481
**IQ2**	0.526	0.595	0.474	**0.821**	−0.127	0.563	0.457	0.434
**IQ3**	0.580	0.523	0.468	**0.812**	−0.219	0.488	0.464	0.469
**IQ5**	0.556	0.568	0.478	**0.837**	−0.169	0.576	0.466	0.472
**LCA1**	−0.337	−0.231	−0.049	−0.179	**0.920**	−0.303	−0.165	−0.286
**LCA2**	−0.358	−0.295	−0.140	−0.206	**0.929**	−0.327	−0.245	−0.324
**LCA3**	−0.370	−0.287	−0.045	−0.152	**0.907**	−0.326	−0.190	−0.266
**PU1**	0.716	0.688	0.367	0.576	−0.345	**0.883**	0.565	0.628
**PU2**	0.688	0.752	0.415	0.548	−0.305	**0.922**	0.535	0.523
**PU3**	0.707	0.748	0.426	0.570	−0.303	**0.921**	0.526	0.508
**PU4**	0.730	0.772	0.414	0.591	−0.309	**0.908**	0.535	0.609
**SInf1**	0.490	0.525	0.496	0.472	−0.234	0.486	**0.901**	0.374
**SInf2**	0.509	0.540	0.518	0.499	−0.204	0.525	**0.928**	0.411
**SInf3**	0.545	0.567	0.434	0.446	−0.153	0.532	**0.844**	0.353
**SInf4**	0.555	0.592	0.568	0.606	−0.178	0.553	**0.856**	0.456
**SQ1**	0.532	0.547	0.357	0.463	−0.291	0.534	0.420	**0.863**
**SQ2**	0.559	0.593	0.378	0.488	−0.227	0.505	0.372	**0.872**
**SQ3**	0.637	0.617	0.363	0.552	−0.312	0.596	0.404	**0.890**

**Fig. 4:**
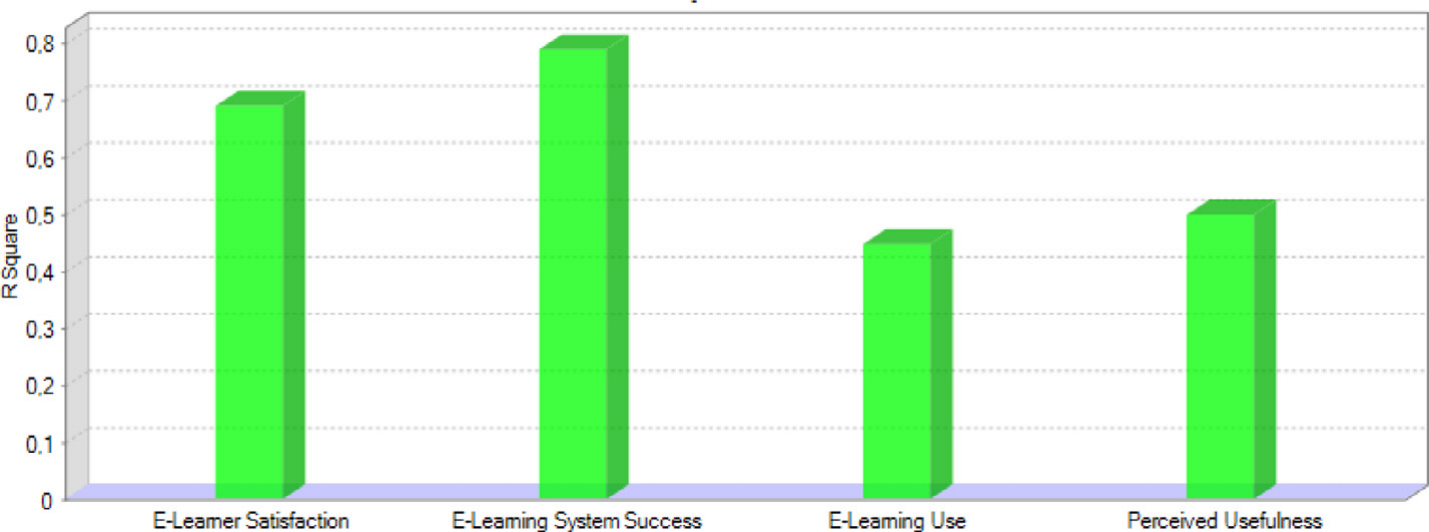
Coefficient of determination of the endogenous constructs- Output SmartPLS.

As indicated in Fig. 4, the values of the coefficient of determination of the couple endogenous constructs; perceived usefulness, and e-learning system use are moderated, which are 0.499 and 0.447 respectively. In addition, the values of R² of the e-learner satisfaction, and e-learning system success are substantial, which are 0.690 and 0.789 respectively.

The size effect (f^2^) values are all acceptable, except the effect of system quality and perceived usefulness on e-learning systems use (Table 9). The system quality and perceived usefulness have no significant effect size on e-learning system use (f^2^ <0.02).

**Table 9:** Effect size.

Constructs		f^2^	Signification
**System Quality**	Perceived Usefulness	0.207	Medium effect size
	E-Learning System Use	0.004	No effect size
	E-Learner Satisfaction	0.074	Small effect size
**Instructor Quality**	Perceived Usefulness	0.218	Medium effect size
	E-Learning System Use	0.149	Small effect size
	E-Learner Satisfaction	0.066	Small effect size
**Social Influence**	E-Learning System Use	0.123	Small effect size
**Learner Computer Anxiety**	E-Learner Satisfaction	0.035	Small effect size
**Perceived Usefulness**	E-Learning System Use	0.002	No effect size
	E-Learner Satisfaction	0.374	Large effect size.
	E-Learning System Success	0.249	Medium effect size
**E-Learning System Use**	E-Learning System Success	0.129	Small effect size
**E-Learner Satisfaction**	E-Learning System Success	0.372	Large effect size.

The predictive relevance (Q^2^) values are all greater than zero, which makes it possible to conclude that the model has an acceptable predictive power [14]. Finally, the Goodness of Fit of the Model of this study is very strong (GoF = 0,674,868 > 0.36) [16].

**Fig. 5:**
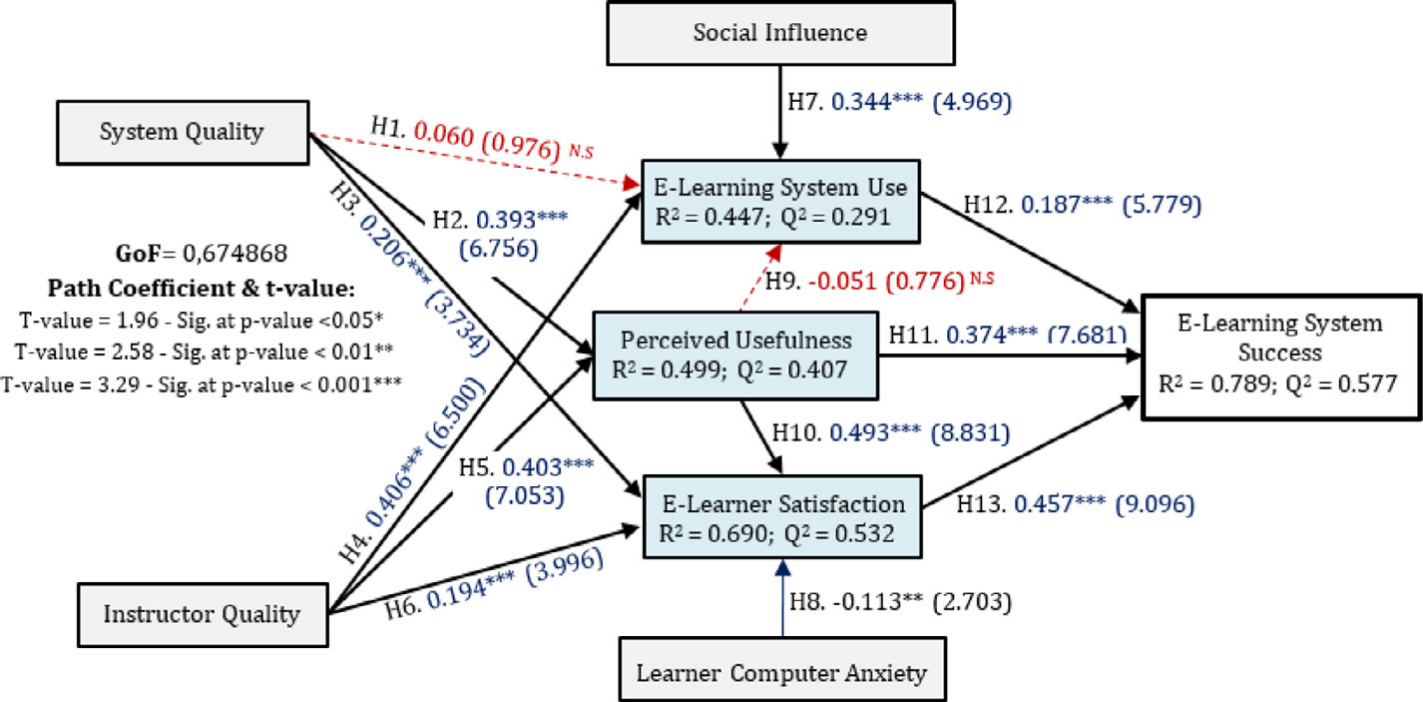
Structural equation model analysis.

According to SmartPLS outputs, it turns out that instructor quality contributes to the explanation of perceived usefulness, e-learning systems use, and e-learner satisfaction. Likewise, the system quality has a positive and significant effect on perceived usefulness, and e-learner satisfaction. On the other hand, social influence has a significant effect on e-learning systems use. In the same, the perceived usefulness contributes to the explanation of e-learner satisfaction. In contrary, learner computer anxiety has a significant and negative effect on e-learner satisfaction. Finally, the perceived usefulness, e-learning systems use, and e-learner satisfaction greatly contributes to the explanation of e-learning system success (Fig. 5).

## Ethics Statement

The consent of respondents was obtained. Participation in the study was voluntary, and participants could withdraw from the survey at any point. The online survey was completely anonymous and does not contain any information allowing identifying the participant.

## CRediT Author Statement

**Abdelaziz Ouajdouni**: Conceptualization, Methodology, Software, Data curation & Analysis, Formal analysis; **Omar Boubker**: Writing - Original draft preparation, Investigation, Reviewing and Editing; **Khalid Chafik**: Supervision, Project Administration.

## Declaration of Competing Interest

The authors declare that they have not known competing financial interests or personal relationships, which have, or could be perceived to have, influenced the work reported in this article.
